# The mediating/moderating role of cultural context factors on self-care practices among those living with diabetes in rural Appalachia

**DOI:** 10.1186/s12889-021-11777-7

**Published:** 2021-10-02

**Authors:** Brittany L. Smalls, Adebola Adegboyega, Ellen Combs, Matthew Rutledge, Philip M. Westgate, Md. Tofial Azam, Felipe De La Barra, Lovoria B. Williams, Nancy E. Schoenberg

**Affiliations:** 1 Department of Family and Community Medicine, College of Medicine, University of Kentucky, 2195 Harrodsburg Road, Suite 125, Lexington, KY 40504 USA; 2 Center for Health Equity Transformation, College of Medicine, University of Kentucky, 372 Healthy Kentucky Building, Lexington, KY 40536 USA; 3 Department of Statistics, College of Arts and Science, University of Kentucky, 725 Rose Street, Multidisciplinary Science Building 0082, Room 303, Lexington, KY 40536 USA; 4 Department of Biostatistics, College of Public Health, University of Kentucky, 725 Rose Street, MDS 205, Lexington, KY 40536 USA; 5 University of Kentucky College of Medicine, William R. Willard Education Building, MN 150, Lexington, KY 40536 USA; 6 College of Nursing, University of Kentucky, 751 Rose Street, 539 CON, Lexington, KY 40536 USA; 7 Department of Behavioral Science, College of Medicine, University of Kentucky, Medical Center, MN 150, Lexington, KY 40536 USA

**Keywords:** Social support, Religiosity, Self-care, Rural Appalachia

## Abstract

**Background:**

The aim of this study was to examine whether cultural factors, such as religiosity and social support, mediate/moderate the relationship between personal/psychosocial factors and T2DM self-care in a rural Appalachian community.

**Methods:**

Regression models were utilized to assess for mediation and moderation. Multilevel linear mixed effects models and GEE-type logistic regression models were fit for continuous (social support, self-care) and binary (religiosity) outcomes, respectively.

**Results:**

The results indicated that cultural context factors (religiosity and social support) can mediate/moderate the relationship between psychosocial factors and T2DM self-care. Specifically, after adjusting for demographic variables, the findings suggested that social support may moderate the effect of depressive symptoms and stress on self-care. Religiosity may moderate the effect of distress on self-care, and empowerment was a predictor of self-care but was not mediated/moderated by the assessed cultural context factors. When considering health status, religiosity was a moderately significant predictor of self-care and may mediate the relationship between perceived health status and T2DM self-care.

**Conclusions:**

This study represents the first known research to examine cultural assets and diabetes self-care practices among a community-based sample of Appalachian adults. We echo calls to increase the evidence on social support and religiosity and other contextual factors among this highly affected population.

**Trial registration:**

US National Library of Science identifier NCT03474731. Registered March 23, 2018, www.clinicaltrials.gov.

## Background

Type 2 diabetes mellitus (T2DM) is a major public health burden, with approximately 34.2 million adults (about 10.5% of the population) in the United States (US) diagnosed with T2DM [1]. As of 2018, Kentucky ranks 7th in the nation for T2DM with a prevalence of 13.8%, which has more than doubled in rate from 2000 [2]. Adding to this burden, T2DM disproportionately affects vulnerable populations, including adults over 65 years [1], ethnic and racial minorities, and those who reside in rural areas [1]. Within the US rural population, nearly one in five (17%) adults in rural Appalachia have been diagnosed with T2DM [2]. Rural Appalachians are disproportionately likely to experience factors that predispose them to the development of T2DM, such as higher levels of stress, obesity, food insecurity, as well as low levels of health literacy and limited access to health services [3, 4]. In addition to these predisposing factors, rural dwellers are exposed to challenging social-environment factors, including high rates of poverty and sparse community and medical services, that may complicate the management of T2DM. Individuals living with T2DM are at-risk for numerous complications, including diabetic retinopathy, nephropathy, neuropathy, cardiovascular disease, amputations, and premature death [4]. To attain optimal health outcomes, persons with diabetes must attend multiple physician visits per year; adhere to several different types of medications; engage in many facets of self-care, including home glucose monitoring, healthy eating, and exercise; and negotiate barriers to management, such as cost of care while balancing work and life commitments [5].

Moreover, it is important to recognize the strengths and cultural values of Appalachia when examining these complex health issues. Throughout the region, Appalachian people exhibit strong ties to community, especially family and extended family [6]. These relationships can be a source of social support when encountering difficulties. Additionally, members of rural Appalachian communities may place value on being careful and responsible with one’s resources [7]. One of the most prominent features of Appalachian culture is a strong belief in independence and individualism, which may have its roots in the early environments of the people who settled in the Appalachian Mountains [6]. In order to survive in oftentimes-harsh conditions it was vital that families learned to be self-reliant. Modern-day challenges such as poverty and limited medical services may have reinforced these values in contemporary Appalachia.

Previous research indicates that cultural context factors, such as social support and religiosity, influence engagement with T2DM self-care activities. Social support is characterized as a multi-dimensional phenomenon that refers to membership and participation in voluntary associations as well as formal and informal relationships among significant others, associates, and colleagues [8]. Because most of the self-care of diabetes occurs at home, family members are likely one of the most important source of social support. An observational study among African Americans found that social support is associated with T2DM-related quality of life and self-care practices [9]. Other studies have also shown that individuals with greater social support followed recommended self-care practices such as dietary recommendations and increasing physical activity [10, 11]. Studies examining the role of perceived social support in T2DM self-care practices found that social support is multifaceted in the lives of patients with T2DM [12]. Not only has strong social support correlate with an increased quality of life and improved self-care behaviors, social support helped the patient cope with a stressful chronic disease [13]. Contrarily, some literature show that some cultural norms may contradict T2DM management. For instance, close family relationships may present as a barrier to T2DM management [14]. In addition, one study found that, while social support may increase overall well-being, there were no significant relationships between social support and HbA1c [15]. Given that T2DM management can be complex and an ongoing struggle [16], there is need to understand how cultural context factors influence T2DM self-care management of vulnerable populations, such as those living in rural Appalachia.

Interactions with local organizations such as faith-based institutions and community centers can also serve as a source of social support and influence self-care practices among individuals with T2DM [17]. An estimated 72% of Americans are affiliated with a religion [18], thereby providing an extended social network for most individuals who attend places of worship. Faith-based institutions can serve as partners in the development and implementation of health programs, given that they involve close social relationships, have an existing infrastructure, and play pivotal roles in the community [19]. Religiosity may also serve as a coping mechanism in response to perceived stress, and individuals who identify as religious or spiritual report better self-perceived health and life satisfaction [20]. Furthermore, studies have indicated correlations between religion and social support, which has a positive association with managing chronic illness. Whether it is through an additional coping strategy, an outlet for strength and comfort, or complementary to active therapy, religiosity has shown to provide positive outcomes with diabetes management [21].

Though most existing literature suggests that social support and religiosity may influence T2DM self-care, there is a paucity of research that assesses these cultural context factors among rural Appalachians. To develop meaningful interventions that promote behavior change in this vulnerable population, it is important to assess the influence religiosity and social support have on T2DM self-care. Therefore, the aim of this study was to examine whether cultural factors, such as religiosity and social support, mediate/moderate the relationship between personal/psychosocial factors and T2DM self-care in a rural Appalachian community.

## Methods

### Study overview

This paper reports the results of baseline cross-sectional data collected as part of the ongoing study “Community to Clinic Navigation to Improve Diabetes Outcomes” (R01 DK112136, PI: Schoenberg). The baseline data collection included a diverse array of behaviors (e.g., self-care behaviors) and domains (e.g., social support, religiosity) relevant to optimal HbA1c levels [20]. Data collection included interviews that lasted between 45 and 80 min. Study approval was obtained by the Office of Research Integrity at the University of Kentucky.

### Conceptual model

**Fig. 1:**
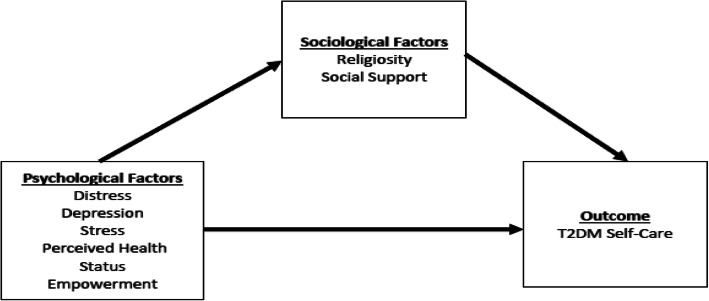
Conceptual Model for Analysis Adapted from Biopsychosocial Model

In this paper, we present an analysis to determine if cultural context factors (social support, religiosity) mediate/moderate the relationship between psychosocial factors and T2DM self-care practices. This analysis was informed by the biopsychosocial model which suggests that on an individual level, the interdependence of psychology, sociology (e.g, cultural context factors), and biology determine health outcomes. We have broadly used the biopsychosocial model to inform this current analysis (see Fig. 1).

### Study setting

The project is currently taking place in six counties in rural Appalachian Kentucky. These counties were selected because of their high rates of T2DM and longstanding collaboration with community partners, such as churches, health care facilities, and community centers [22]. All of the counties are considered by the Appalachian Regional Commission to be economically distressed, with high rates of unemployment, poverty, and low income [23].

### Recruitment

Participants were recruited through community sites, including churches and senior centers. Community recruitment offers advantages (over clinical recruitment) including: enrolling hard to reach individuals with impeded access to clinics, avoiding selection bias of healthier participants better able to access clinics, increasing comfort and trust of participants and ensuring that the facilities are accessible to participants after hours [24]. Participants were eligible to participate if they were 18 years or older, live in Appalachia Kentucky with no plans to relocate out of the area in the next 18 months and showed a willingness and ability to participate (i.e., no major cognitive impairment), and had a diagnosis of T2DM and/or HbA1c levels at least 6.5%. Individuals interested and potentially eligible were asked to complete written informed consent and undergo point-of-care HbA1c screening to confirm eligibility. With the high prevalence of undetected T2DM in Appalachian communities, all interested individuals at elevated risk of T2DM (as determined by the American Diabetes Association Risk Test, with a score of ≥2) were screened for eligibility [25]. Given close-knit rural communities and the likelihood that household members tend to attend church or community centers together, more than one member of a household was eligible to participate.

### Measures

#### Independent variables

The independent variables were measured using the following validated instruments: problem areas in managing T2DM (5-item Problem Areas in Diabetes Scale) [26], diabetes distress (Diabetes Distress Scale) [24], empowerment (Diabetes Empowerment Scale) [27], health status (SF36) [28], depressive symptoms (Center for Epidemiologic Studies Depression Scale) [29], and stress (Perceived Stress Scale) [30].

#### Potential mediators/moderators (cultural context variables)

Religiosity was assessed by asking two questions: *“How often do you attend church or other religious meetings (more than once a week, once a week, a few times a week, a few times a year, once a year or less, and never)?”* and *“How often do you spend time in private religious activities, such as prayer, private meditation, or Bible study (more than once a day, daily, two or three times a week, once a week, a few times a month, rarely or never)?”* Social support was measured using 4 scales found in the Patient-Reported Outcomes Measurement Information System (PROMIS) including the subscales of companionship, informational support, ability to participate in social roles and activities, and emotional support [31]. PROMIS has been shown to be valid and reliable irrespective of disease [32].

#### Outcome variable

We used the Summary of Diabetes Self-Care Activities (SDSCA) to assess key self-management components, including overall diet, fruit/vegetable, and fat consumption; exercise; blood sugar self-testing; and footcare. These scores were then averaged to create an overall measure of self-care [33].

#### Demographic variables

In the analysis the following variables were included in the adjusted mediator/moderator models: age, sex, race/ethnicity, marital status, education level, employment status, insurance status, financial status. In addition, diabetes knowledge measured using the Diabetes Knowledge Questionnaire and the number of chronic conditions were determined using the following question: “Has a doctor, nurse, or other health professional ever told you that you had any of the following?” The responses included heart attack, coronary heart disease/angina, stroke, kidney disease, high blood pressure, high cholesterol, type 2 diabetes, type 1 diabetes, and Hepatitis C.

### Statistical analysis

Regression models were utilized to assess for mediation and moderation. Multilevel linear mixed effects models and GEE-type logistic regression models were fit for continuous (social support, self-care) and binary (religiosity) outcomes, respectively. Within the multilevel linear mixed effects models, random site effects, and random household effects within sites, are used to account for the possibility of multiple levels of clustering due to the study design. Due to the high prevalence of religiosity, accounting for such clustering in the GEE-type logistic regression models often results in non-convergence, and therefore a working independence structure with Kauermann and Carroll (2001) [34] bias-corrected standard errors are utilized in order to ensure valid inference. To obtain standardized beta coefficients, continuous outcome and predictor variables were centered and standardized. All tests were two-sided. Statistical significance was defined as *p* < 0.05. Analyses were conducted in SAS version 9.4 (SAS Institute, Cary, N.C.).

## Results

The study sample included 356 participants from 26 community sites in rural Appalachia Kentucky. Sample demographic characteristics are described in Table 1. The mean HbA1c was 7.7 and 45.2% had two or more chronic conditions. Consistent with local demographics, the majority of the sample were White (98%). Most participants were married (54.4%), women (64.6%), insured (98%) and approximately two-thirds (67.6%) had earned more than a high school diploma. In addition, 42.1% were retired and 28.9% indicated that they sometimes struggled to make ends meet.

**Table 1:** Baseline Participant Characteristics (*N* = 365)

Variable	Mean ± SD or N (%)	N Missing
Age	64.2 ± 10.6	3
Gender		0
Women	230 (64.6%)	
Men	126 (35.4%)	
Race/Ethnicity		0
White	349 (98.0%)	
African American	7 (2.0%)	
Hemoglobin A1c	7.7 ± 1.7	28
Marital Status		4
Married	208 (58.4%)	
Divorced	55 (15.4%)	
Never Married	21 (5.9%)	
Widowed	68 (19.1%)	
Education		0
HS/GED	115 (32.3%)	
Associates	43 (12.1%)	
Some College	61 (17.1%)	
Bachelor	24 (6.7%)	
Graduate/Professional	113 (31.7%)	
Employment		0
Full-time	63 (17.7%)	
Part-time	12 (3.4%)	
Homemaker	49 (13.8%)	
Disabled	73 (20.5%)	
Retired	150 (42.1%)	
Unemployed	9 (2.5%)	
Insurance Status		0
Insured	349 (98.0%)	
Uninsured	7 (2.0%)	
Financial Status		8
Have more than you need to live well	91 (25.6%)	
Have just about enough to get by	154 (43.3%)	
Sometimes struggle to make ends meet	103 (28.9%)	

Table 2 displays the results of the psychosocial and cultural context factors among the sample population. For each of the measures, a higher score indicated that participants indicated affirmative responses. The mean scores for each psychosocial factor were as follows: perceived stress was 21.8 ± 9.3 (range 0–47), diabetes distress was 28.3 ± 12.2 (range 17–83), empowerment 31.7 ± 7.0 (range 8–40), problem areas in diabetes 7.7 ± 1.7 (range 5.3–14), and self-reported health status 47 ± 21.6 (range 0–95). In addition, 69.4% of participants indicated no depressive symptoms. As for measures of cultural context factors, the mean social support score was 114.7 ± 17.0 (51–150) and 13.8% reported not participating in any religious activities.

**Table 2:** Personal and Psychosocial Characteristics of Study Participants (*N* = 356)

Variables	Mean ± SD or N(%)	N Missing
Stress	21.8 ± 9.3	37
Distress	28.3 ± 12.2	22
Empowerment	31.7 ± 7.0	10
Social Support	114.7 ± 17.0	2
Diabetes Knowledge	15.3 ± 3.9	0
Problem Areas	7.7 ± 1.7	13
Diabetes Self-Care	17.1 ± 6.3	1
Health Status	47.0 ± 21.6	0
Health Conditions in addition to T2DM		15
0	17 (4.8%)	
1	72 (20.2%)	
2	161 (45.2%)	
3	58 (16.3%)	
4	24 (6.7%)	
5	7 (2.0%)	
6	2 (0.6%)	
Depressive Symptoms		19
Yes	90 (25.3%)	
No	247 (69.4%)	
Religiosity		
Yes	170 (47.8%)	
No	185 (52.0%)	

For each independent variable, regression models were conducted to determine if social support and/or religiosity had a mediating/moderating role on T2DM self-care. These analyses were designed to evaluate how these cultural context factors may influence how T2DM-related psychosocial factors influence health outcomes. Table 3 shows the results from models adjusting age, gender, marital status, education, employment, financial status, insurance, smoking status, and health conditions.

**Table 3:** Adjusted Model Assessing Cultural Context Factors as Mediators/Moderators between Psychosocial Characteristics and T2DM Self-Care (*N* = 356)

Outcome	Mediation: Step 1		Mediation: Step 2				Mediation: Step 3		Moderation	
	Self-Care		Religiosity		Social Support		Self-Care		Self-Care	
**Depression**										
Effect	Estimate (SE)	*p*-value	Estimate (SE)	*p*-value	Estimate (SE)	*p*-value	Estimate (SE)	*p*-value	Estimate (SE)	*p*-value
Intercept	0.162 (0.494)	0.743	−0.524 (1.185)	0.662	0.119 (0.479)	0.805	−0.754 (0.639)	0.239	−0.833 (0.640)	0.194
Depressed	− 0.198 (0.140)	0.157	−0.813 (0.345)	0.100	0.050 (0.136)	0.714	−0.163 (0.141)	0.248	0.036 (0.171)	0.835
Religiosity							0.209 (0.118)	0.078	**0.334 (0.132)**	**0.012**
Social Support							−0.013 (0.058)	0.819	− 0.012 (0.076)	0.870
Depressed * Religiosity									**−0.522 (0.255)**	**0.042**
Depressed * Social Support									0.000 (0.120)	0.998
**Distress**										
Intercept	0.027 (0.470)	0.954	−0.960 (1.164)	0.418	0.180 (0.475)	0.704	−0.024 (0.469)	0.959	−0.017 (0.463)	0.971
Distress	**−0.398 (0.061)**	**<.0001**	−0.267 (0.155)	0.159	−0.083 (0.062)	0.186	**−0.389 (0.061)**	**<.0001**	**−0.275 (0.075)**	**0.000**
Religiosity							**0.221 (0.112)**	**0.049**	**0.224 (0.111)**	**0.045**
Social Support							−0.049 (0.057)	0.394	− 0.089 (0.060)	0.135
Distress * Religiosity									**−0.271 (0.113)**	**0.017**
Distress * Social Support									0.081 (0.046)	0.081
**Empowerment**										
Intercept	0.176 (0.479)	0.713	−0.950 (1.177)	0.427	0.254 (0.472)	0.591	0.112 (0.479)	0.815	0.057 (0.486)	0.907
Empowerment	**0.216 (0.057)**	**<.001**	0.023 (0.137)	0.877	0.089 (0.056)	0.114	**0.215 (0.057)**	**<.001**	0.143 (0.082)	0.083
Religiosity							**0.240 (0.115)**	**0.038**	**0.242 (0.115)**	**0.036**
Social Support							−0.053 (0.057)	0.354	−0.054 (0.060)	0.368
Empowerment * Religiosity									0.137 (0.112)	0.222
Empowerment * Social Support									−0.002 (0.046)	0.974
**Stress**										
Intercept	0.192 (0.501)	0.701	−0.719 (1.177)	0.547	0.146 (0.480)	0.761	0.136 (0.503)	0.786	−0.096 (0.503)	0.849
Stress	**−0.244 (0.074)**	**0.001**	−0.275 (0.173)	0.253	0.017 (0.071)	0.811	**−0.227 (0.075)**	**0.003**	−0.120 (0.095)	0.206
Religiosity							0.184 (0.122)	0.131	0.199 (0.120)	0.099
Social Support							0.002 (0.062)	0.978	−0.099 (0.074)	0.182
Stress * Religiosity									−0.186 (0.124)	0.135
Stress * Social Support									**0.184 (0.077)**	**0.017**
**Self-Reported Health Status**										
Intercept	0.071 (0.484)	0.884	−0.835 (1.158)	0.478	0.235 (0.465)	0.614	0.010 (0.484)	0.984	−0.142 (0.490)	0.773
Health Status	0.112 (0.065)	0.089	**0.508 (0.165)**	**0.037**	−0.089 (0.064)	0.162	0.086 (0.066)	0.197	0.033 (0.089)	0.707
Religiosity							0.228 (0.117)	0.051	**0.230 (0.116)**	**0.049**
Social Support							−0.028 (0.058)	0.634	−0.051 (0.060)	0.398
Health Status * Religiosity									0.102 (0.113)	0.366
Health Status * Social Support									−0.100 (0.062)	0.109

*Bold indicates statistical significance of *p* < 0.05; *SE* Standard Error

### Depressive symptoms

Depressive symptoms were not a significant predictor of self-care (β = − 0.198, SE = 0.140, *p* = 0.157), religiosity (β = − 0.964, SE = 0.492, *p* = 0.051), or social support (β = 0.050, SE = 0.136, *p* = 0.714) after adjusting for demographic variables. Thus, there is no evidence that either religiosity or social support are mediators for depressive symptoms or their effects on self-care after adjusting for demographic variables. Neither interaction covariate is statistically significant, and therefore we do not have strong enough evidence to conclude that either religiosity or social support moderate the impact of depressive symptoms on self-care.

### Diabetes distress

Diabetes distress was a significant predictor of self-care (β = − 0.398, SE = 0.061, *p* < 0.0001) after adjusting for demographic variables. Although distress was a significant predictor of religiosity (β = − 0.790, SE = 0.232, *p* = 0.001), it was not a significant predictor of social support (β = − 0.083, SE = 0.062, *p* = 0.186) after adjusting for demographic variables. When adjusting for religiosity, social support, and demographic variables, distress remains a significant predictor of self-care (β = − 0.395, SE = 0.063, *p* < 0.0001) with an estimated association that is only negligibly changed, thus providing no support of mediation. The interaction effect of distress and religiosity was statistically significant (β = − 0.348, SE = 0.129, *p* = 0.007), indicating that social support may moderate the effect of stress on self-care even after adjusting for demographic variables.

### Empowerment

Empowerment was a significant predictor of self-care (β = 0.216, SE = 0.057, *p* < 0.001) after adjusting for demographic variables. However, empowerment was not a significant predictor of either religiosity (β = 0.215, SE = 0.204, *p* = 0.295) or social support (β = 0.089, SE = 0.056, *p* = 0.114) after adjusting for demographic variables. Thus, there is no evidence that either religiosity or social support are mediators for empowerment on the effect of self-care after adjusting for demographic variables. After adjusting for religiosity, social support, and demographic variables, empowerment remained a significant predictor of self-care (β = 0.211, SE = 0.057, *p* < 0.001). No statistically significant moderations were observed.

### Perceived stress

Stress was a significant predictor of self-care (β = − 0.244, SE = 0.074, *p* = 0.001) after adjusting for demographic variables. However, stress was not a significant predictor of either religiosity (β = − 0.634, SE = 0.388, *p* = 0.103) or social support (β = 0.017, SE = 0.071, *p* = 0.811) after adjusting for demographic variables. Thus, there is no evidence that either religiosity or social support are mediators for stress on the effect of self-care after adjusting for demographic variables. When adjusting for religiosity, social support, and demographic variables, stress was still a significant predictor of self-care (β = − 0.221, SE = 0.075, *p* = 0.004). Moreover, the interaction effect of stress and social support was statistically significant (β = 0.195, SE = 0.077, *p* = 0.012), indicating that social support may moderate the effect of stress on self-care even after adjusting for demographic variables.

### Self-reported health status

Health status was not a significant predictor of self-care after adjusting for demographic variables (β = 0.112, SE = 0.065, *p* = 0.089). In addition, health status was a significant predictor of religiosity (β = 0.604, SE = 0.213, *p* = 0.005) but not of social support (β = − 0.089, SE = 0.064, *p* = 0.162). As such, there was not strong enough evidence to conclude there was an association to mediate. Furthermore, no statistically significant moderations were observed.

### Summary of key findings

In summary, the results indicated that cultural context factors (religiosity and social support) can mediate/moderate the relationship between psychosocial factors and T2DM self-care. Specifically, after adjusting for demographic variables, the findings suggest that social support may moderate the effect of depressive symptoms and stress on self-care. Religiosity may moderate the effect of distress on self-care, and empowerment is a predictor of self-care but is not mediated/moderated by the assessed cultural context factors. When considering health status, religiosity is a moderately significant predictor of self-care and may mediate the relationship between perceived health status and T2DM self-care.

## Discussion

We aimed to examine the role played by cultural context, defined by social support and religiosity, in influencing diabetes self-care practices in a sample of rural Appalachians. Such insights are particularly essential for those populations experiencing the greatest health inequities for several reasons. First, it was essential to understand the experience of T2DM among those populations that are disproportionately affected and oftentimes overlooked in research. Second, an improved understanding of local assets, including traditions of social support and religiosity, that can be leveraged to improve self-care ensures that programs and approaches will be culturally acceptable. Third, from an economic perspective, knowing whether and how cultural assets affect health behaviors provided insights into potentially cost effective and sustainable interventions. Our examination of whether and how cultural context factors influence engagement with T2DM self-care among rural resident highlighted several key findings.

First, our results suggested a lack of a direct effect of social support on self-care behaviors. This finding is not necessarily surprising—while social connections generally confer a positive connotation, extensive research shows that individuals close to a person may actually undermine health [35, 36]. It is unclear precisely why social support or connections may not enhance (and may even undermine) self-care practices; some literature suggests that social support from people who are struggling with the same challenges—lower income, fewer resources, less education- may reinforce suboptimal self-care behaviors. Alternatively, participants may feel a broad overall sense of social support, but that such support may not necessarily be applied to specific behaviors. For example, a participant may feel able to glean health information from friends and family but may lack the specific information on optimal T2DM dietary intake. Thus, the mere perceived availability of such support may not actually translate into information that is useful to improve self-care.

While social support was not directly associated with self-care practices, social support did appear to moderate the relationship between depressive symptoms and stress as related to self-care. Such a finding is consistent with existing literature that demonstrates that standard social support domains like emotional, informational, and tangible support can diminish the negative consequences of distress and depression [24, 37]. For example, it is possible that simply having the perception that one’s social network has supportive resources may buffer the negative effects of stress on self-care practices and health outcomes.

Although the factors driving these associations remains unresolved, our results indicating an association between religiosity and self-care are consistent with those of most researchers [38, 39]. Researchers have speculated that religiosity may foster optimal self-care practices by encouraging positive psychological orientation and a sense of purpose that emphasize upholding religious laws and tenets (e.g., body as a temple), by providing a community of support and trust, and by increasing the potential receipt of health information in a trusted environment [20, 40].

Our findings reveal that religiosity may moderate the effect of distress on self-care. It is plausible that higher levels of religiosity diminish distress by offering a stronger sense of purpose, perspective, and connectedness. Greater connection to a higher power may reduce the sense of helplessness and distress people feel and empower them to engage in recommended self-care behaviors. Indeed, in this study, empowerment was predictive of self-care, although it was not mediated or moderated by the cultural context factors of social support and religiosity. This finding reinforces others that have determined that when persons with T2DM feel empowered, they are better able to engage in optimal self-care practices [41, 42]. Empowerment involves a sense of responsibility for undertaking actions that affect health. Numerous studies, including interventions, have demonstrated that enhancing this sense of empowerment leads to a greater sense of health ownership and successful negotiation of self-care.

We acknowledge that this Appalachian sample may not reflect the experiences of rural residents overall or the general population of those with T2DM. Specifically, reflecting the local demographics, our sample was predominantly White. Recruitment of study participants from churches may have increased selection bias regarding religiosity. Therefore, those who were recruited had a propensity to be religious, which may have influenced the analysis. Twice as many women were enrolled. In addition, like all self-reported data, we were not able to verify self-care practices. Additionally, we employed a limited assessment of religiosity and spirituality that may only assess particular aspects of religiosity. We acknowledge that these constructs are complex and involve multiple dimensions. Since we were unable to undertake a more comprehensive assessment of religiosity and spirituality, we also were unable to discern the precise role this construct plays in self-care behavior. In order to determine a more precise role, comparing moderate/mediate factors between religiosity and non-religiosity participants may provide insight into how religiosity may influence self-care behavior. Finally, although we focused on two of the most frequently mentioned assets in the Appalachian context, social support and religiosity, there are likely many others that we overlooked. For example, we did not examine resilience, a psychosocial construct known to be associated with behavior and described as a cultural asset of Appalachian residents. The dataset we employed had not collected data on resilience; however, in the future, such a focus is warranted. Lastly, this paper is a secondary analysis of the primary study and the measures used were limited by the aims of the study.

## Conclusion

Despite limitations, this study represents the first known research to examine cultural assets and diabetes self-care practices among a community-based sample of Appalachian adults. Over the past several decades, researchers have demonstrated that social context affects behavioral, clinical, and psychosocial outcomes among people with T2DM. While many of these findings suggest that social support and religiosity tend to be beneficial, other research disconfirms these findings, indicating the need for additional insights. Though there is literature indicating that social support and religiosity may influence T2DM self-care, there is a paucity of literature that assesses these cultural context factors in rural Appalachia. Moreover, to develop meaningful interventions that promote behavior change in this vulnerable population, we have identified the influence religiosity and social support has on T2DM self-care. Thus, immediate next steps are to increase the evidence on social support and religiosity and other contextual factors among this highly affected population. This can take many forms including, community-based and community-engagement interventions, utilizing community stakeholders (e.g., faith-based leaders, informal community leaders) to cultivate behavior change, and use implementation science to tailor existing evidence-based, community and social support interventions to facilitate behavior change in the target population.

## Data Availability

The data used and/or analyzed during the current study are available from the corresponding author on reasonable request. The datasets generated and/or analyzed during the current study are not publicly available since this is an ongoing study and only baseline data was used in the current analysis; however, the datasets are available from the corresponding author on reasonable request.

## Abbreviations

T2DM: Type 2 diabetes
GEE: Generalized estimating equations
SE: Standard error
SDSCA: Summary of Diabetes Self-Care Activities
PROMIS: Patient Reported Outcomes Measurement Information System
